# Current realities versus theoretical optima: quantifying efficiency and sociospatial equity of travel time to hospitals in low-income and middle-income countries

**DOI:** 10.1136/bmjgh-2019-001552

**Published:** 2019-08-21

**Authors:** Kerry LM Wong, Oliver J Brady, Oona Maeve Renee Campbell, Christopher I Jarvis, Andrea Pembe, Gabriela B Gomez, Lenka Benova

**Affiliations:** 1 Infectious Disease and Epidemiology, London School of Hygiene and Tropical Medicine Faculty of Epidemiology and Population Health, London, UK; 2 Centre for Mathematical Modelling for Infectious Diseases, London School of Hygiene and Tropical Medicine, London, United Kingdom; 3 Obstetric and Gynaecology, Muhimbili University of Health and Allied Sciences, Dar es Salaam, United Republic of Tanzania; 4 Global Health and Development, London School of Hygiene and Tropical Medicine, London, London, UK; 5 Public Health, Institute of Tropical Medicine, Antwerpen, Belgium

**Keywords:** physical accessibility to health services, health services research, health service provision, health equity, health inequality, shortest travel time, sub-Saharan Africa

## Abstract

**Background:**

Having hospitals located in urban areas where people, resources and wealth concentrate is efficient, but leaves long travel times for the rural and often poorer population and goes against the equity objective. We aimed to assess the current efficiency (mean travel time in the whole population) and equity (difference in travel time between the poorest and least poor deciles) of hospital care provision in four sub-Saharan African countries, and to compare them against their theoretical optima.

**Methods:**

We overlaid the locations of 480, 115, 3787 and 256 hospitals in Kenya, Malawi, Nigeria and Tanzania, respectively, with high-resolution maps of travel time, population and wealth to estimate current efficiency and equity. To identify the potential optima, we simulated 7500 sets of hospitals locations based on various population and wealth weightings and percentage reallocations for each country.

**Results:**

The average travel time ranged from 38 to 79 min across countries, and the respective optima were mildly shorter (<15%). The observed equity gaps were wider than their optima. Compared with the best case scenarios, differences in the equity gaps varied from 7% in Tanzania to 77% in Nigeria. In Kenya, Malawi and Tanzania, narrower equity gaps without increasing average travel time were seen from simulations that held 75%–90% of hospitals at their current locations.

**Interpretations:**

Current hospital distribution in the four sub-Saharan African countries could be considered efficient. Simultaneous gains in efficiency and equity do not necessarily require a fundamental redesign of the healthcare system. Our analytical approach is readily extendible to aid decision support in adding and upgrading existing hospitals.

**Key questions**

**What is already known?**

- The populations is typically heterogeneously distributed in space. The efficiency of healthcare provision can be optimised by choosing populous locations, especially in densely-populated urban cities, as health facility sites. This generally minimises average travel time, thus meeting the efficiency objective; but leaves long travel times for the rural and often poorer population and decreases equity.
- Especially in low-resource settings, the healthcare system is often challenged by the tension of balancing the efficiency and equity objectives.

**What are the new findings?**

- Through quantifying the efficiency and equity of travel time to hospital in Kenya, Malawi, Nigeria and Tanzania, we found that current spatial distribution of hospital in these countries was close to optimally efficient, but tended to be inequitable and pro-rich, and often unnecessarily so.
- We showed that the current spatial distribution of hospital in most countries is 75%–90% similar to the hypothetical optimised scenarios.

**What do the new findings imply?**

- While it is unrealistic to imagine moving hospitals, our analytical approach can readily be extended to aid decision support in placing new health facilities and upgrading existing ones while optimising the efficiency and equity objectives.
- Maximising total beneficiaries and accounting for those in hard-to-reach areas can be balanced, and achieving gains in both priorities does not necessarily involve a fundamental redesign of the healthcare system.

## Background

Health services are often provided in relation to population density for efficiency and other economic and political reasons described below. More recently, interests in ensuring equitable access and achieving universal health coverage (UHC)—the aspiration that all people obtain access to the health services they need without risking financial hardship—had grown. While the concept of equitable access is multifaceted, most studies measure the equity of use of key services; nevertheless, physical accessibility remains a fundamental consideration in many low-income and middle-income countries (LMICs) and may be a key factor underpinning inequitable service use. It would be useful to have a tool that assesses both efficiency and equity of existing service locations. This could ultimately be used to identify options which compensate for inequitable physical locations, including by adding facilities or in which locations to best upgrade facilities.

A good level of physical accessibility is attained when healthcare is available and located within reasonable reach to people. Physical accessibility is often expressed as the travel time or distance between healthcare and the population.^1^ Service provision strategies that aim to optimise physical accessibility are most efficient when the population’s average travel time is minimised for a given total provision cost.^2^ Selecting densely populated urban cities to locate health facilities is a good way to achieve the efficiency objective.

Two determinants of hospital locational preference related to population and population density are the economy and politics.^3^ Healthcare providers tend to locate in areas with good markets. The private sector is a case in point, but in the public decision-making process of establishing a new hospital or renovating an existing one, location also plays an important role specifically with regard to guaranteeing the profit return on investment.^4 5^ The impact of politics on the spatial distribution of healthcare is also recognised. Friedmann examined the effect of geopolitics, observing that power is concentrated in the capital cities and to a lesser extent in provincial headquarters.^6^ Such concentration makes urban locations the locus of political power and the homes of the elite, who often have mechanisms working to influence the process of locational and allocational decision-making to their advantage.^7 8^ In Nigeria, for instance, it has been argued that the location of public facilities could be affected by reasons including community monetary contributions and political considerations, such as when a commissioner or minister influences the selection of their home-town in health facility site selection.^9^ The net effect of all this is that privileged people and places are better served, and rural and remote places less populated, poorer and with worse physical access to health services. Such unequal opportunities to accessing health services can exacerbate existing inequalities in healthcare utilisation, with the marginalised and vulnerable faring the worst.

In most LMICs, governments aim to meet the health needs of those living in rural and hard-to-reach areas with health centres and health posts. If such facilities are considered, then average travel time are reduced, and equity improved. However, many of these facilities only provide outpatient care,^10–12^ and the fuller range of life-saving health services—caesarean section, treatment of postpartum haemorrhage, emergency operations, specialised therapies, gynaecology/paediatric inpatient care, just to name a few—are generally only available in hospital settings.^10–12^ Health service provision assessments, such as Service Provision Assessment or Service Availability and Readiness Assessment, also often demonstrate that health centres and health posts are underequipped and understaffed for the basic functions that they are expected to perform.^13–15^ Equitable distribution of higher-level care is therefore paramount to ensure accessibility to a broad range of services; yet the challenge of which lies in a relatively small effective geographic coverage for the high-cost professionals, equipment and interventions required.

Inequity of physical access to surgical care and emergency obstetric care (services usually provided only in hospital settings) in poorer areas/subpopulations has been shown suboptimal compared with wealthier areas/subpopulations in LMICs in a few national and subnational studies.^16–18^ However, there is still a distinct lack of nationally-representative and generalisable studies in the literature. This calls for a better understanding of the trade-off between efficiency and equity intrinsic to public decision-making for higher-level care. The aim of this study is to develop an approach to examine the balance between efficiency and equity of physical accessibility to hospital in four LMICs in sub-Saharan Africa. We calculate the current levels of efficiency and equity, and compare them to their theoretical maxima realised through a simulation exercise. Future applications of our approach could be used to decide where best to upgrade or add facilities.

## Data and methods

### Study settings

We studied four LMICs in sub-Saharan Africa—Kenya, Malawi, Nigeria and Tanzania. These countries were selected because they had a complete georeferenced listing of hospitals (master facility list, MFL) and were variable in terms of demography, geography, healthcare financing and health service delivery. National statistics are presented in table 1.

**Table 1:** Country data and statistics in 2016 (unless otherwise stated)

	Kenya	Malawi	Nigeria	Tanzania
Total area (km^2^)^43^	580 367	118 484	923 768	947 300
% land area^43^	98	79	99	94
National population (million)^43^*	47	18	181	54
% urban population^43^*	26	16	48	32
Gini Index^44^†	48	44	49	38
GDP per capita, purchasing power parity (current US$)^43^*	3020	1159	6039	2653
Health expenditure per capita, purchasing power parity (current US$)^43^	157	108	215	97
% out-of-pocket	33	11	72	26
% external	18	54	10	37
% birth registration coverage^45^	67	67	30	26
Number of hospital beds per 10 000 population^45^	14	13	5	7
% population >2 hour travel time to public emergency hospital care^30^	7	7	8	25

*Data from 2015.

†Data from 2013.

GDP, gross domestic product.

### Data

We used five sources of data in this study: MFL, population, friction, Demographic and Health Survey (DHS) and country administrative areas boundary data. MFL data were obtained online.^19–23^ All hospitals with recorded geographic coordinates within the corresponding national extents were included (online supplementary A). Hospitals were classified according to the respective MFLs. As the Tanzanian MFL did not include hospitals in Zanzibar, this subregion was excluded. We also excluded one hospital on Likoma Island in Malawi.

10.1136/bmjgh-2019-001552.supp1 Supplementary data

We used the Gridded Population of the World population estimate (version 4) for every 1×1 km^2^ non-overlapping pixel across the study countries.^24^ The Malaria Atlas Project’s land surface friction file for all land pixels between 85° north and 60° south for a nominal year 2015 was used to enumerate land-based travel speed.^25^ The friction value, given as the time (in minutes) needed to travel one metre, represents the generalised difficulty of crossing a pixel depending on factors such as types of road, water bodies and terrain with slopes.^25^ In the study region, the minimum and maximum friction values to travel 1 meter were 0.0005 min and 0.3 min—equivalent to travelling at the speed of 120 km/hour and 0.2 km/hour—respectively.

We estimated median household wealth index—a composite measure of a household’s cumulative living standard—with DHS data as means of assessing pixel-level wealth. Wealth index was modelled based on a suite of covariates—population density, day-time land surface temperature and vegetation index, elevation, potential evapotranspiration, aridity index and night-time light emission. To derive high-resolution poverty maps, we used model-Based Geostatistics for Kenya and a generalised additive model for each of Malawi, Nigeria and Tanzania. Our choices of modelling methods were based on a previous analysis that compared the performances of these approaches.^26^ Lastly, we sourced country administrative areas boundary files from the freely available Database of Global Administrative Area (www.gadm.org).^27^

### Calculating travel time to the nearest hospital, efficiency and equity

For each country, travel time to the nearest hospital was computed for every 1×1 km^2^ pixel using an algorithm that Weiss and colleagues devised to identify the path that requires the least time through the friction surface between two points.^25^ The application of the algorithm on the friction surface to construct an accessibility map enumerating travel time to the nearest hospital has been performed in a previous study.^28^ Once travel time to the nearest hospital from all pixels was obtained, we superimposed data on population count and estimated wealth to produce estimates of the average travel time to the nearest hospital (time_all_) and the same for just the poorest and richest 10% of pixels—time_poor_ and time_rich_—respectively.

### Conceptual and operational definitions

The definition of efficiency in economics is mainly as allocative efficiency and pertains to the optimal distribution of resources for maximum production of health, while technical efficiency refers to the production of health at minimum costs.^29^ In this study, we defined efficiency as a function of the average travel time (time_all_) to the nearest hospital across the population by country, assuming maximum access to care will facilitate the maximum production of health. Optimal efficiency was attained when time_all_ was minimised. On the other hand, equity is often considered in terms of systematic differences that are unjust or unfair, implying a value judgement.^29^ For the purpose of this study, we looked at equity from a distributional perspective alongside socioeconomic status, with the aim of achieving equal access to available care for equal need. Our operational definition of equity is then measured as the excess in travel time of the poorest decile compared with the richest decile (time_poor_ – time_rich_). Optimal equity is characterised by a minimum absolute value of the equity gap—equal physical access to the nearest hospital regardless of the status of wealth. Our calculation and optimisation are summarised in table 2. We compared the observed average travel time and the observed equity gap against their optimal values.

**Table 2:** Study metrics and optimisations

Metrics definition	
Time_all_	Average travel time to the nearest hospital in the population
Time_poor_	Average travel time to the nearest hospital for the poorest 10% of pixels
Time_rich_	Average travel time to the nearest hospital for the richest 10% of pixels
**Optimi** **s** **ations**	
Efficiency	min(time_all_)
	Minimise overall travel time to the nearest hospital in the population
Equity gap	min(abs(time_poor_ – time_rich_))
	Minimise absolute difference between travel time to the nearest hospital for the poorest and for the richest decile

### Identifying optimising efficiency and equity through a simulation

For each country, we identified the optimal levels for efficiency and equity using a simulation approach. Conditioned on the observed number of hospitals, and excluding all unpopulated places, we simulated hospital sites from every 1×1 km^2^ non-overlapping pixel within the study region. We sampled hospitals locations with five weighting schemes—probability directly proportional to population count and the square of population count, wealth index, inverse of wealth index and unweighted. These weights were chosen based on their potentials to optimise average travel time and the equity gap. We anticipated that, for instance, weighting by population count should minimize average travel time, i.e.,optimise overall efficiency. Moreover, we relocated three different proportions of current hospital sites. The first batch of simulations relocated a random sample of 10% of hospitals from the current MFL using each of the five weights with 500 replicates. The second batch relocated a random 25% of hospitals, and the third with all 100% hospitals relocated. This totalled 7500 simulated scenarios for each study country (500 replicates×5 probabilistic weighting schemes×3 relocation proportions). We calculated time_all_, time_poor_, time_rich_ and the equity gap for each simulated scenarios, and identified the best cases that optimised efficiency and equity. Conditioned on not increasing the observed time_all_, we then identified the best simulation for minimising the absolute value of the equity gap to determine the potential of improving equity of physical access without compromising efficiency.

We also conducted the same analysis using only government/public hospitals, since provision of private sector care may not be efficiency or equity driven, data are likely more credible and complete for public sector facilities, and private hospitals are likely to be particularly unaffordable to the least wealthy subpopulation.

### Patient and public involvement statement

We did not involve patients or the public in our work.

## Results

### Current spatial distribution of hospitals, optimal efficiency and optimal equity

**Figure 1:**
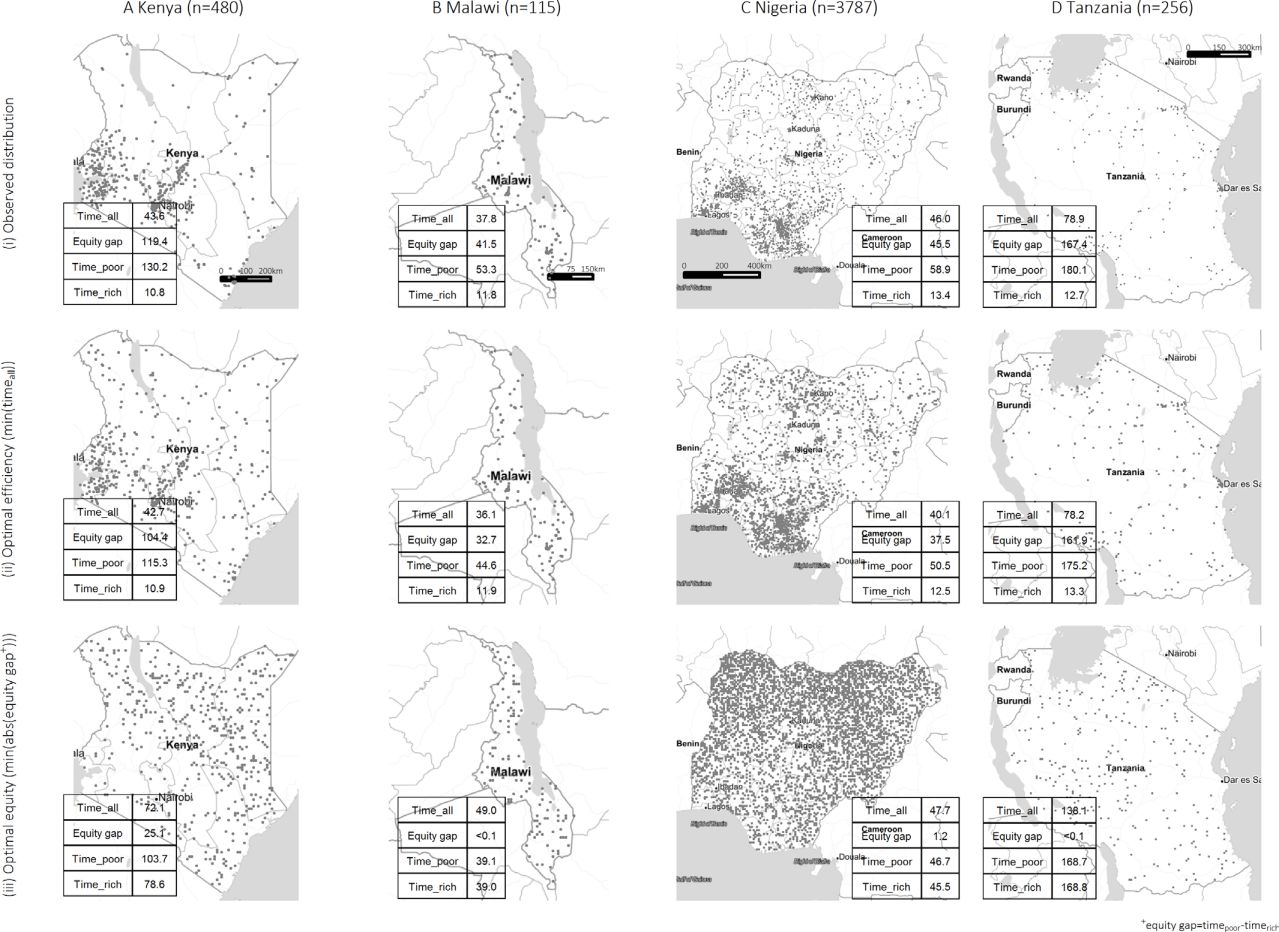
Observed and simulated hospital locations and travel time to the nearest hospital (in minutes).

The observed spatial distributions of hospitals in Kenya (n=480), Malawi (n=115), Nigeria (n=3787) and Tanzania (n=256) are presented in figure 1(i)A–D. The observed average travel times to the nearest hospital (time_all_) were 44, 38, 46 and 79 min for the four countries. In Kenya, for instance, average travel time ranged from 11 min for the richest 10% decile to 130 min for the poorest 10% decile (figure 1(i)A)—an equity gap of 119 min. The observed equity gaps of Malawi, Nigeria and Tanzania were 42, 46 and 167 min, respectively.

Maps of simulated hospital locations resulting in optimal efficiency are shown in figure 1(ii)A–D. In Kenya, the most efficient simulation resulted in time_all_ of 43 min (figure 1(ii)A), 2% better than the observed. Time_poor_ of the most efficient simulation for Kenya was 115 min, 11% less than the observed 130 min. The percentages of reduction in time_all_ comparing the observed distribution against the most efficient simulations for Malawi, Nigeria and Tanzania were 4, 13 and 1, respectively; and time_poor_ of these most efficient simulations were also lower than their respective observed values.

Figure 1(iii)A–D illustrates simulated hospitals locations with optimal equity (minimum absolute value of time_poor_-time_rich_). The equity gap of the most equitable simulation for Kenya was 25 min—79% narrower than the observed. However, time_all_ increased to 72 min and time_rich_ to 79 min. In the other three countries, the equity gaps could almost be fully eliminated, and increases in both time_all_ and time_rich_ from their observed values were also seen.

Online supplementary B shows time_all_ and the equity gap of the observed spatial distributions of public hospitals in Kenya, Malawi, Nigeria and Tanzania. Similar comparisons were seen when these observed values were compared with their respective optima.

10.1136/bmjgh-2019-001552.supp2 Supplementary data

### More efficient and more equitable

**Figure 2:**
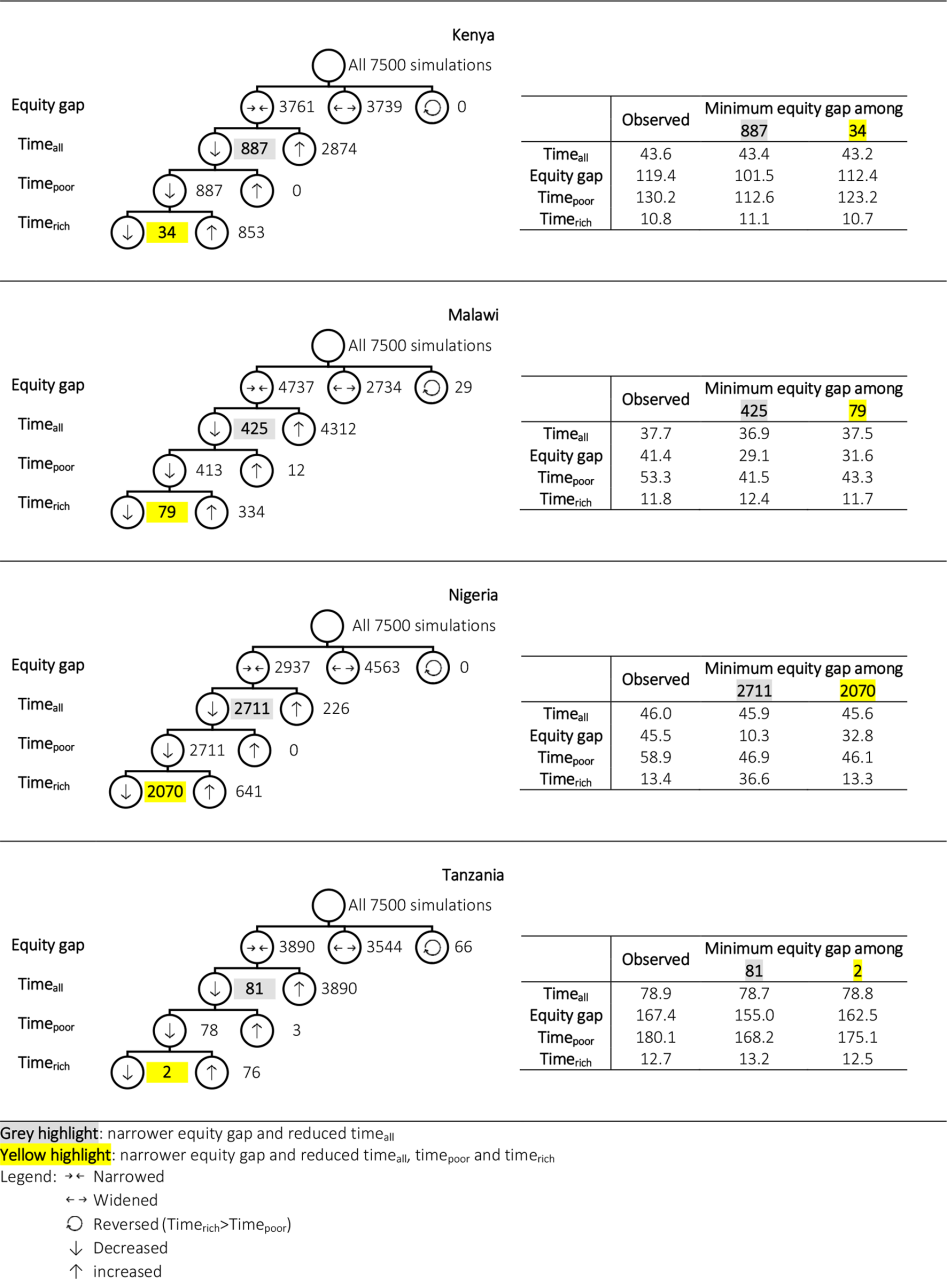
Summary of simulation results in minutes (hospitals in all sectors).

Figure 2 summarises the results of all 7500 simulations for each study country. The numbers of simulation that decreased time_all_ (more efficient) and narrowed the equity gap (more equitable) were 887 for Kenya, 425 for Malawi, 2711 for Nigeria and 81 for Tanzania. Restricting to these subsets which improved equity without compromising on efficiency, the most equitable simulations for Malawi and Nigeria, for instance, narrowed the equity gaps to 29 min (30% reduction) and 10 min (77% reduction), respectively. The associated increase in time_rich_; compared with the observed was small in Malawi (and also in Kenya and Tanzania), but was more substantial for Nigeria (from 13 to 37 min). Lastly, 34, 79, 2070 and 2 simulations remained for Kenya, Malawi, Nigeria and Tanzania, respectively, when further conditioned on not increasing time_rich_.

**Figure 3:**
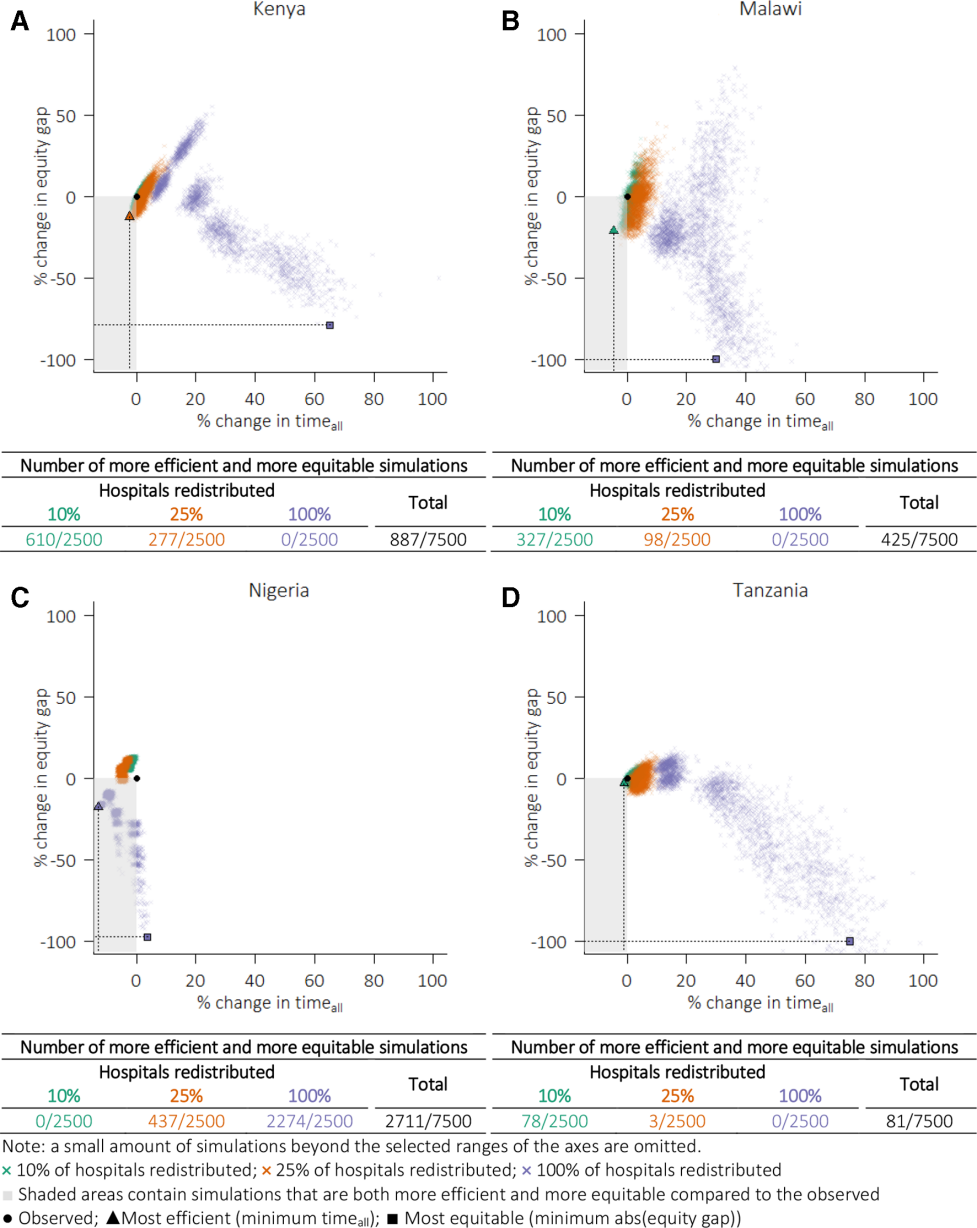
Relative changes in equity gap and average travel time comparing the observed from simulation results.

The 887 simulations with reduced time_all_ and the equity gap for Kenya are shown in the lower-left quadrants (grey area) in figure 3. In Kenya, Malawi and Tanzania, redistributing 10%–25% of hospitals (or holding 75%–90% of hospitals at their current locations) accounted for all those simulations that decreased time_all_ and narrowed the equity gap. In Nigeria, however, simulations that relocated 100% of hospitals accounted for majority (2274 of 2711, or 84%) of those simulations that were more efficient and more equitable (figure 3).

## Discussion

### Summary

We conducted a multicountry simulation study to examine the trade-offs between efficiency (average travel time) and sociospatial equity (absolute difference in travel time between the poor and least poor deciles) of physical access to the nearest hospital in Kenya, Malawi, Nigeria and Tanzania. As means of assessing current system performance, we compared current efficiency and equity with their theoretical optima, obtained through a simulation exercise that relocated hospitals and thus provided alternative values for efficiency and equity.

Across the study countries, the average travel time was very close to their respective theoretical optima, but the observed travel time for the least poor tended to be too high for optimal efficiency. Compared with the observed, the best cases for efficiency for Kenya, Malawi and Tanzania were only mildly better (<5%); the best case for Nigeria was 13% more efficient. In all countries but Kenya, the equity gaps in travel time could almost be completely closed, although this would have required the whole population, and especially those living in the least poor places, to travel for longer. Without compromising efficiency, we still found simulations with narrower equity gaps. The potential extent of equity gap reduction varied across countries from being almost negligible in Tanzania to being prominent in Nigeria. Furthermore, simultaneous improvements in efficiency and equity were found from simulations that held 75%–90% of hospitals at their current locations for Kenya, Malawi and Tanzania, while more substantial reorganisation involving up to 100% of hospitals were required in Nigeria.

### Strengths and limitations

Empirical research of health service provision across key dimensions of inequality have not been widely conducted due to the lack of suitable data. To our knowledge, this is the first multicountry study to quantify the trade-offs between the conflicting goals of hospital care provision efficiency and sociospatial equity, and to identify current areas of substandard performance in sub-Saharan Africa. Using the Malaria Atlas Project’s Friction Surface 2015, we were able to refine the scale of results and generalisability compared with previous studies conducted at the county and sub-district levels.^16–18^

Our results have important implications but should be interpreted in light of their limitations. First, our theoretical optimisation approach did not account for key factors of the possibility and practicability of hospital care provision, including physical environment and the supporting infrastructure. Second, enlisting, georeferencing and validating MFL data require an extensive effort and are prone to error. When compared with Ouma and colleagues’ inventory of public emergency hospital care delivery points, we noted certain mismatches in numbers—for example, 399 public hospitals in Kenya from Ouma *et al* 30 against 390 as obtained from the MFL used in this analysis. These discrepancies may be partially due to different inclusion criteria, such as the provision of emergency services at a facility. We did not make consolidating the whole list a high priority as the small differences found would unlikely affect our results substantially; however, validity check and data completeness assessment might be relevant in future work where manual checking of facilities becomes a feasible task. Third, travel times were derived by varying speeds of travel based on land cover characteristics and topography, and travel time estimates (and wealth estimates) assumed homogeneity among individuals located in the same pixel. Perceived physical barriers, actual travel pattern, road conditions, time spent in transit and so on likely vary among people living in the same pixel and are unaccounted for. Other assumptions of travel friction (eg, the impact of seasonal and temporal variabilities) and wealth (eg, displacement of DHS geocodes) have been detailed elsewhere.^25 26^ Lastly, our definition for hospital was based on data on the type of health facility as given in the MFLs; and these hospitals may vary somewhat in capacity, quality of care and the range of health services that they provide.

### Service provision efficiency versus equity

Equity in health service provision seeks a distribution so that everyone has a ‘fair’ opportunity to access healthcare, and that no sociodemographic subgroup is disadvantaged and left behind. In this study, equity was defined as equal travel time to the nearest hospitals regardless of geographic location and wealth. When compared with the actual geographic locations of hospitals, the best simulated cases for equity across the study countries all had greater numbers of hospitals locating in poorer places, and smaller numbers of hospitals locating in richer places. Because wealth and people typically concentrate at the same places and poverty in others, average travel time on the whole would increase, and overall efficiency compromised.

Compromising efficiency to redress inequity is a distributional issue with geographic, economic and political dimensions.^31^ However, without notably increasing average travel time, or reducing efficiency, we still found ‘excess’ hospital care provision in richer locations in all study countries from our simulation. The equity corrections identified relative to the observed mildly curtail physical accessibility for the rich and shortened travel time for the poor; thereby improving overall access and narrowing the equity gap simultaneously. For Kenya, Malawi and Tanzania, a small difference in the current system involving 10%–25% of hospitals in simulated alternative hospitals locations resulted in gains in both efficiency and equity. Such a small difference indicates the closeness between the current system and the theoretical best scenario. In Nigeria, however, the equity correction involves relocating a larger proportion of hospitals (from south to north, qualitatively). We observed similar results in our sensitivity analysis with public-sector hospitals only, so current equity gap does not appear to exist solely because private healthcare providers concentrate in more profitable populated, urban and rich places. Possible explanations are that Nigeria has too few numbers of hospitals in the north with respect to the number of people supposed to benefit from such services, and somewhat populous settlement patterns in semi-urban places, where sizeable populations are affected by suboptimal physical accessibility. Gaps in service coverage studied here may partially be filled by the provision of pre-hospital care at lower-level facilities and a high-functioning referral system, but in settings such as those studied here, lower-level facilities are often limited in their capacity to manage sick individuals and effectively refer complicated cases upwards, thus rendering people’s chances to healthcare utilisation to meet their health needs.^32–34^ Expanding the provision of care without consideration of its quality and adequacy to meet the needs of the target population may create a fragmented system that stratify people into tiered benefits.^35^

In this study, we assessed how close the current distribution of hospitals is to the theoretical best scenarios. While existing hospitals are unlikely to be relocated, our simulations help quantify the best cases for efficiency and equity with provision fixed at its current level. The national health sector plans in many LMICs overtly display their commitment to advancing service provision/delivery at both the national and subnational levels as means of moving towards achieving UHC and access to safe, effective, quality and affordable essential health services for all.^36–39^ Potential policy approaches to increase access may involve building better road networks and strengthening interfacility communication and transportation services. Building new hospitals is also an attractive project for governments and international funders^40^; and strategies to ensure their integration with the existing system and overall functioning are essential.^40^ In LMICs, where the physical and human resources needed to sustain the structure of a comprehensive healthcare system are limited, decision-makers may be interested in the spatial decision mechanism developed here as it provides insights into where a hospital might be added/upgraded to improve existing levels of efficiency and equity. The method presented in this study can be used to support such spatial decision-making by conditioning on all existing hospitals and simulating new locations. The efficiency-equity balance might be particularly pertinent to emergency health services planning due to the critical role of travel time. Some solutions also extend to a hierarchy of health facilities of varying levels,^41^ and are suitable for ensuring the population’s accessibility to a whole network of both primary healthcare and the wider range of other health services.^40^

A holistic approach to equitable healthcare provision should also take into consideration the rapid population and economic shifts. Urbanisation, secondary cities development and internal migration (rural to urban) amplify the ever-increasing healthcare demand in populated and urban areas. Moreover, rural places are often characterised by topographic and logistical constraints, where adequate healthcare provision requires investment in basic infrastructure (roads, water and sanitation networks, electricity grid) that is difficult for the health sector to finance alone. In many LMICs, there has only been slow to modest progress in meeting the health needs of ‘everyone, everywhere’.^42^ The preference to perform efficiently, and the maximisation of total beneficiaries, over equity concerns has also been explicated in some policy-making arenas.^42^ This raises concerns around structuring the health system so that no one is left behind; and unless health interventions are designed to promote equity, movement towards UHC may lead to improvements at the national level while continuing to exclude the marginalised by reinforcing existing distributional imbalance. This is of particular importance to countries with a low overall access quotient.^30^ Analysis similar to that presented in this paper, when a country-geocoded MFL becomes ready to a country, can play an integral role in making sure that the provision of all types and levels of services in local areas are not left behind from the effort of making national improvements.

## Conclusion

Our results suggest that the goal of bringing hospitals within the reach of the largest proportion of the population is satisfactorily attained. This is probably due to the commendable effort to prioritise healthcare in populated areas. However, current hospital distribution falls short to be equitable, with the health needs of those living in remote and hard-to-reach places most neglected, especially since they also tend to have greater unmet needs of healthcare. A separate set of criteria for establishing new hospitals in scarcely populated and poor places may be needed, despite expected lower cost-effectiveness of such locations compared with urban locales. Our results suggest the possibility of dual optimisation of efficiency and sociospatial equity compared with the current system. Encouragingly, achieving the two goals simultaneously does not necessarily require a fundamental redesign of the current system, and strategies to optimising placement of new facilities are available to help drive future decisions.
