# Supplementary material for: Current realities versus theoretical optima: quantifying efficiency and sociospatial equity of travel time to hospitals in low-income and middle-income countries
Source: BMJ Glob Health. 2019 Aug 21;4(4):e001552. doi: 10.1136/bmjgh-2019-001552 (PMC6730570; doi:10.1136/bmjgh-2019-001552)
Supplement: Supplementary data [file bmjgh-2019-001552supp001.pdf]

Supplementary A. Facility data from Master Facility List (MFL)

| | Kenya | Malawi | Nigeria | Tanzania |
|---|---|---|---|---|
| Number of facilities | 9430 | 977 | 33850 | 7783 |
| Number of hospitals | 485 | 116 | 3787 | 265 |
| Number of hospital per 1,000 km$^2$ land area | 0.85 | 1.23 | 4.16 | 0.30 |
| Number of hospital per 10,000 population | 0.10 | 0.07 | 0.21 | 0.05 |
| Number of hospitals with no geographic coordinates | 5 | 0 | 0 | 9 |
| Number of hospitals included in the main analysis | 480 | 115[+] | 3787 | 256 |
| Number of public hospitals included in the main analysis | 390 | 50 | 1244 | 119 |

[+] One hospital on Likoma Island was excluded from the analysis.

Number of hospitals per 10,000 population by first administrative division

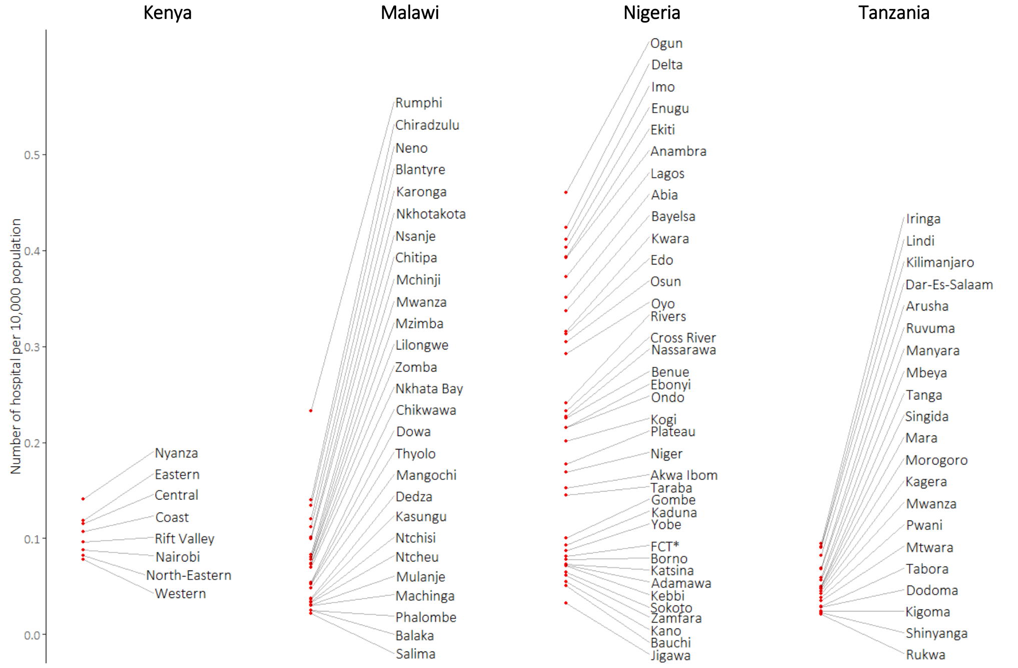


*FCT = Federal Capital Territory
