# Supplementary material for: Current realities versus theoretical optima: quantifying efficiency and sociospatial equity of travel time to hospitals in low-income and middle-income countries
Source: BMJ Glob Health. 2019 Aug 21;4(4):e001552. doi: 10.1136/bmjgh-2019-001552 (PMC6730570; doi:10.1136/bmjgh-2019-001552)
Supplement: Supplementary data [file bmjgh-2019-001552supp002.pdf]

Supplementary B. Observed and simulated hospitals locations and travel time to the nearest hospital (in minutes)

		All hospitals					Public hospitals only				
			$Time_{all}$	Equity gap	$Time_{poor}$	$Time_{rich}$		$Time_{all}$	Equity gap	$Time_{poor}$	$Time_{rich}$
Kenya	Observed		43.6	119.4	130.2	10.8		43.9	118.8	130.2	11.4
	Most efficient (min $time_{all}$)		42.7	104.4	115.3	10.9		43.2	110.5	122.0	11.5
	Most equitable (min(abs(equity gap))	(n=480)	72.1	25.1	103.7	78.6	(n=390)	79.5	24.0	108.7	84.7
	Pro-poor (min($time_{poor}$))		70.2	26.5	101.0	74.5		69.0	68.0	106.2	38.3
	Pro-rich (min($time_{rich}$))		44.6	123.4	133.9	10.5		44.6	123.7	134.8	11.1
Malawi	Observed		37.7	41.4	53.3	11.8		44.0	52.3	66.3	13.9
	Most efficient (min $time_{all}$)		36.1	32.7	44.6	11.9		42.5	37.9	51.9	13.9
	Most equitable (min(abs(equity gap))	(n=115)	49.0	<0.1	39.1	39.0	(n=50)	70.4	<0.1	56.5	56.5
	Pro-poor (min($time_{poor}$))		52.9	-3.8	37.8	41.6		63.1	3.8	45.5	41.7
	Pro-rich (min($time_{rich}$))		38.5	41.5	53.0	11.5		45.5	49.7	62.4	12.8
Nigeria	Observed		46.0	45.5	58.9	13.4		48.3	44.7	60.1	15.5
	Most efficient (min $time_{all}$)		40.1	37.5	50.0	12.5		47.5	50.6	61.7	11.1
	Most equitable (min(abs(equity gap))	(n=3787)	47.7	1.2	46.7	45.5	(n=1244)	63.6	0.1	60.2	60.1
	Pro-poor (min($time_{poor}$))		46.0	32.0	45.8	13.9		61.8	26.5	58.9	32.4
	Pro-rich (min($time_{rich}$))		45.1	49.9	59.0	9.1		49.3	52.1	63.1	10.9
Tanzania	Observed		78.9	167.4	180.1	12.7		92.3	183.6	198.4	14.8
	Most efficient (min $time_{all}$)		78.2	161.9	175.2	13.3		90.6	186.1	201.5	15.3
	Most equitable (min(abs(equity gap))	(n=256)	138.1	<0.1	168.7	168.8	(n=119)	168.7	0.1	198.2	198.1
	Pro-poor (min($time_{poor}$))		129.5	55.7	150.2	94.5		168.1	96.8	183.8	87.0
	Pro-rich (min($time_{rich}$))		83.3	174.1	186.3	12.1		96.9	188.0	201.4	13.4
